# Exploring non-alcohol-based disinfectant: virucidal efficacy of arginine and Zinc chloride against feline calicivirus

**DOI:** 10.3389/fmicb.2025.1550295

**Published:** 2025-02-13

**Authors:** Marcella Kong Li Ying, Srdan Masirevic, Yong Wah Tan, Jan K. Marzinek, Stephen John Fox, Chandra S. Verma, Peter J. Bond, Yoshiki Ishida, Jiquan Liu, Chun Song Chua, Justin Jang Hann Chu

**Affiliations:** ^1^Procter & Gamble (Singapore), Singapore, Singapore; ^2^Bioinformatics Institute, Agency for Science, Technology and Research, Singapore, Singapore; ^3^Collaborative Translation Unit for HFMD, Institute of Molecular and Cell Biology, Agency for Science, Technology and Research (A*STAR), Singapore, Singapore; ^4^Department of Biological Sciences, Faculty of Science, National University of Singapore, Singapore, Singapore; ^5^Infectious Disease Translation Research Programme, Yong Loo Lin School of Medicine, National University of Singapore, Singapore, Singapore; ^6^Laboratory of Molecular RNA Virology and Antiviral Strategies, Department of Microbiology and Immunology, Yong Loo Lin School of Medicine, National University of Singapore, Singapore, Singapore

**Keywords:** feline calicivirus, arginine, Zinc chloride, disinfectant, molecular modeling

## Abstract

**Introduction:**

Norovirus, a leading cause of acute gastroenteritis worldwide, is notably stable in the environment due to its non-enveloped nature. In the absence of effective vaccines or treatments, disinfection remains the primary prevention strategy, highlighting the importance of virucidal efficacy in household care products. Conventional effective disinfectants are predominantly alcohol-based, but alcohol is known to pose health risks, such as skin irritation. This study investigates a non-alcohol-based alternative, specifically a combination of Arginine and Zinc chloride (ZnCl_2_).

**Methods:**

Utilizing MS2 bacteriophage as a surrogate, we identified a robust combination of arginine and ZnCl_2_ that is effective against Feline Calicivirus (FCV), a mammalian virus surrogate model for Norovirus.

**Results:**

Our results determined a 5 min contact time at pH 11 as optimal, achieving significant virucidal activity against FCV without pH-induced reversibility. Dynamic Light Scattering (DLS) and transmission electron microscopy (TEM) analyses suggested that the mechanism of action for the Arg-Zn^2+^-Arg complex does not involve capsid disruption. Further insights from molecular modeling studies revealed that the complex potentially inhibits FCV by occupying a key capsid binding pocket essential for Junctional Adhesion Molecule (JAM) receptor engagement, thereby preventing viral entry.

**Conclusion:**

These findings allow us to propose a novel and non-alcohol-based virucidal approach against viruses from the Caliciviridae family, highlighting the potential of Arg-Zn^2+^-Arg complexes in public health protection.

## 1 Introduction

Since the end of 2019, the entire globe was struck with an unforeseen pandemic caused by SARS-CoV-2, the causative agent of COVID-19 disease. The virus caught much of the world’s population off-guard, overloading healthcare systems which consequently led to a major public health crisis across the world (Mondal and Mitra, 2022). This pandemic highlights the importance of developing novel technologies to safeguard the public against emerging viruses.

Preventing exposure to viruses is one major defensive strategy, in light of the limited availability of specific antiviral treatments (Cheng et al., 2016). Personal hygiene and household care products therefore play an important role in ensuring that the surrounding environment of an individual is uncontaminated. Standardized virucidal testing protocols are hence being continuously developed and used to assess virucidal or antiviral efficacy of disinfectants and sanitizers under realistic conditions (Eggers et al., 2021; Tarka and Nitsch-Osuch, 2021), especially against non-enveloped viruses as they are known to be more resistant to chemicals (Firquet et al., 2015; Mahl and Sadler, 1975).

One commonly tested non-enveloped virus is Norovirus, also known as Norwalk virus. Norovirus is a non-enveloped virus of about 27 nm in size, classified under the *Caliciviridae* family (Robilotti et al., 2015). The virion structure includes 90 dimers of the VP1 capsid protein, forming a T = 3 icosahedral symmetry, along with one or two VP2 proteins (Prasad et al., 1999). VP1 is divided into shell (S) and protruding (P) domains, with the P domains enhancing capsid stability and forming virion protrusions while VP2 protein is believed to be crucial for receptor binding and immune response (Prasad et al., 1999; Tan et al., 2003). It accounts for almost half of all acute gastroenteritis cases worldwide, estimated at about 267 million cases reported each year (Donaldson et al., 2008). Individuals across all ages are susceptible to infection, but with higher mortality in younger and older age groups and in the immunocompromised (Robilotti et al., 2015). Infection occurs thorough the fecal-oral route, and infected individuals experience vomiting and diarrhea, where the virus is shed in the feces and vomitus, which could contaminate surfaces (Kirking et al., 2010; Mary et al., 2011; Nilsson et al., 2003; Nordgren, 2009; Robilotti et al., 2015). Being a non-enveloped virus, Norovirus is extremely stable in the environment, thus contributing to its high transmission rate. Currently there are no effective vaccines or antiviral treatments available for Norovirus infections (Nordgren, 2009), and the most effective way to prevent norovirus infection is through disinfection of fomites. Therefore, due to its threat to public health and chemical resistance, Norovirus is listed in many international standards and frequently used to evaluate virucidal efficacy of chemistries.

Current household disinfectants effective against norovirus use harsh chemical-based solutions such as sodium hypochlorite, quaternary ammonium salts and ethanol. A previous publication has shown that an ethanol-based disinfectant mixed with zinc sulfate presented virucidal efficacy against non-enveloped viruses (Martin-Gonzalez et al., 2020). Nonetheless, frequent exposure to ethanol is known to cause side effects like skin irritation, allergies, and asthma (Suchomel et al., 2009). Hence, there is an ongoing search for non-alcohol-based solutions that are effective against Norovirus.

Arginine, one of the twenty natural amino acids, is also well-known to be a humectant used in cosmetics (Giam et al., 2016; Nenoff et al., 2004). In addition, arginine has previously been reported to affect both protein and lipid interactions (Arakawa and Tsumoto, 2003; Ayako et al., 2006; Sakai et al., 2004; Tsumoto et al., 2005), contributing to its ability to function as a bactericidal and virucidal compound (Cutrona et al., 2015; Ikeda et al., 2012; McCue et al., 2014; Meingast and Heldt, 2020; Mutschler et al., 2016; Sepahi et al., 2017). We explore the virucidal efficacy of combinations of arginine and zinc against viruses, especially non-enveloped Norovirus. Unfortunately, human Norovirus cannot be readily used to assess virucidal efficacy of disinfectants since it cannot be grown in normal cell cultures (Duizer et al., 2004) and require sophisticated systems for successful cultivation (Estes et al., 2019; Jones et al., 2015). Therefore, other culturable viruses from the *Caliciviridae* family, such as feline calicivirus (FCV) and murine norovirus (MNV) are commonly used as surrogates (Cannon et al., 2006; Cromeans et al., 2014; Hoover and Kahn, 1975; Wobus et al., 2006). In addition to mammalian virus surrogates, bacteriophages such as MS2 are also utilized in biosafety level 1 laboratories or in laboratories lacking cell culturing facilities (Dawson et al., 2004; Tung-Thompson et al., 2015). In this study, MS2 served as an initial surrogate to rapidly screen through various concentrations of arginine and ZnCl_2_ solutions to identify the optimal concentration which was subsequently used to challenge FCV and its mechanism of action was determined.

## 2 Materials and methods

### 2.1 Bacterial host and bacteriophages

*Escherichia coli* strain C3000 (ATCC^®^ 15597) was selected as the host for *E. coli* bacteriophage MS2 (ATCC^®^ 15597B1). Firstly, the *E. coli* strain C3000 was streaked onto Luria-Bertani (LB) agar plate and incubated at 37°C for 18–24 h. A single colony was picked and sub-cultured in 20 mL of LB broth and incubated with agitation for another 18–24 h at 37°C. Next, 100 μL of the subculture was inoculated into fresh 20 mL of LB broth and incubated with agitation at 37°C for 4 h, which will be the working bacteria stock where the *E. coli* strain C3000 hosts are at log phase.

MS2 was propagated in the log phase *E. coli* strain C3000. A single colony of MS2 was picked and first resuspended in 1 mL of LB broth before centrifuging at 12,000 rpm for 5 min. The phage supernatant was mixed with 1 mL of log phase *E. coli* strain C3000 and 4 mL of 0.75% (w/v) LB soft agar, before overlaying on top of LB agar. Plates were incubated at 37°C overnight before *E. coli* broth was added and swirled gently for 15 min at room temperature. Subsequently, the *E. coli* broth was collected and centrifuged at 12,000 rpm for 20 min and kept at 4°C until further use.

### 2.2 Cell lines and viruses

Feline calicivirus (Strain F-9; ATCC VR-782) was propagated in Crandell-Rees Feline Kidney (CRFK) cells (ATCC CCL-94). CRFK cells were first cultured at 37°C overnight in minimum essential medium (MEM) supplemented with 1 mM sodium pyruvate, 2 mM of L-glutamate and 10% fetal bovine serum (FBS). During infection, FCV resuspended in MEM + 2% FBS was inoculated onto the CRFK monolayer and incubated at 35°C for 1 h. After incubation, inoculum was removed and fresh MEM + 2% FBS was added before further incubation at 35°C for 16–20 h. Subsequently, cellular supernatant was harvested and stored at −80°C. For dynamic light scattering (DLS) experiments, harvested FCV culture underwent a 0.2 μm filtration before concentration with Centricon Plus 70 (Merck). FCV was further purified and concentrated using sucrose cushion ultracentrifugation at 25,000 rpm at 4°C for 3 h. The virus pellet was resuspended in Tris-NaCl-EDTA (TNE) buffer at pH 8.0 before storage at −80°C.

### 2.3 Virucidal efficacy testing against MS2

A total of 800 μL of test solution was mixed with 100 μL of 3% bovine serum albumin (BSA) (final concentration: 0.3%) and 100 μL of MS2 (1 × 10^9^ pfu/mL). The reaction mix was left to incubate at room temperature for the stipulated contact time before performing 10-fold serial dilution in LB broth. 50 μL of each dilution was added in triplicates onto an *E. coli* strain C3000 overlay that was composed of log phase *E. coli* strain C3000 diluted 10-fold in 0.75% (w/v) LB soft agar, laid on top of LB agar. The petri dishes were incubated at 37°C for 18–24 h and the plaques were counted and expressed as plaques forming units per millimeter (pfu/mL).

### 2.4 Virucidal efficacy testing against FCV

The BS EN 14476:2013+A2:2019 quantitative suspension test under clean conditions was used as a reference for evaluating virucidal efficacy of test solutions against viruses. In summary, 8 parts of the test solution were mixed with 1 part of 3% BSA and 1 part of virus (6–7 × 10^8^ pfu/mL) and incubated at room temperature for the stipulated contact time. Next, 50 μL of the reaction mix was diluted in 450 μL of cold Modified Letheen Broth supplemented with 1.5% Tween 80 and 1% Lecithin (MLBTL). The diluted reaction mix was later serially diluted 10-fold in media before proceeding to plaque assay to determine the concentration of virus left after treatment with the test solution.

### 2.5 Viral plaque assay

Monolayers of CRFK cells in tissue culture plates were washed twice with 1x PBS before inoculation with serially diluted reaction mix in triplicates. Plates were incubated at the virus’s optimum growth temperature for 1 h before rinsing twice with 1x PBS and adding a suitable overlaying media. Plates were further incubated for 48 h to allow plaque formation before fixing with 4% paraformaldehyde and staining with crystal violet. Plaques were counted and expressed as pfu/mL.

### 2.6 Cell viability assay

A total of 8 parts of the test solution were mixed with 1 part of 3% BSA and 1 part of 1x PBS before 10x dilution in cold MLBTL and 10-fold serial dilution in media. Monolayers of cells in 96 well tissue culture plates were inoculated with the serially diluted test solution mix in triplicates and incubated at 35°C for 48 h. Next, the inoculum was replaced with 10% alamarBlue Cell Viability Reagent in its respective media and incubated further at 37°C. Absorbance readings at 570 nm and 600 nm (normalization) were performed and cell viabilities expressed as a percentage with reference to control. Wells with less than 80% viability indicated cytotoxicity.

### 2.7 Dynamic light scattering (DLS)

Concentrated and purified FCV was first diluted 10 times in TNE buffer at pH 8.0 before 0.2 μm filtration to remove viral clumps. Subsequently, 20 μL of FCV was added into 80 μL of test solution and incubated in room temperature for 5 min before 80 μL of reaction mix was loaded into the cuvette. ZS Xplorer software version 3.2.2.5 was used to measure viral particle size using the back scatter angle of detection, and all readings were measured in triplicates at 25°C. The Z-average was associated with the polydispersity index (PI) to estimate the width of the distribution.

### 2.8 Negative stain transmission electron microscopy (TEM)

A total of 1 part of concentrated virus suspension was treated with 4 parts of test solution, incubated at room temperature for 5 min and fixed in 2.5% glutaraldehyde. After fixation, virus was adsorbed onto formvar/carbon film coated copper grid followed by staining with 1% phosphotungstic acid (PTA). Grids were air dried prior to viewing with transmission electron microscope JEM-1400.

### 2.9 Computational modeling

#### 2.9.1 Protein preparation and ligand design

The effect of an Arg-Zn^2+^-Arg ligand on FCV was investigated using a model of the pentameric capsid shell assembly at the 5-fold vertices of the virus (PDBID: 6GHS) (Conley et al., 2019). The model was prepared at pH 11 (a condition relevant for antiviral activity) using the Protein Wizard module of Schrodinger. Concurrently, the ligand complex, consisting of a zinc (Zn^2+^) ion and two arginine (Arg) molecules, was assembled using tools in Schrödinger 2023 Maestro Suites. For adaptation to alkaline conditions (pH = 11), the complex was prepared with the LigPrep module (Johnston et al., 2023), ensuring that correct protonation states were achieved. Subsequently, the geometry of the Arg-Zn^2+^-Arg complex underwent refinement via the Jaguar module, which employs Density Functional Theory (DFT) for quantum mechanical optimization (Bochevarov et al., 2013).

#### 2.9.2 Identification and evaluation of the FCV binding sites

The identification and evaluation of potential binding sites on the prepared pentameric structure of the FCV virus were conducted using the SiteMap tool within the Schrödinger 2023 suite (Halgren, 2009). SiteMap analyzes the protein surface to locate binding pockets, ranking them based on a composite score known as SiteScore. This score reflects a site’s druggability, determined by several key criteria: hydrophobicity, size and shape, polarity, enclosure, solvent exposure and hydrogen bonding potential. Sites with higher SiteScores are considered more druggable, indicating a greater likelihood of successful ligand binding. In this study, the 20 top-ranked binding sites were selected for molecular docking.

#### 2.9.3 Molecular docking and ligand preparation

The LigPrep module within the Schrödinger suite was used to prepare the Arg-Zn^2+^-Arg complex for molecular docking. This preparation step was essential to accurately assign chirality, and generate possible tautomers, ensuring that the ligand was optimally configured for this study. Prior to docking runs, grid generation was carried out based on the coordinates of the top-ranked binding sites identified by SiteMap. Subsequently, the Arg-Zn^2+^-Arg complex was docked into the top-ranked binding sites using the Glide module in Schrödinger (Friesner et al., 2004; Yang et al., 2021). The docking process predicts the preferred orientations of the Arg-Zn^2+^-Arg complex within the binding pocket, and the associated stability of the complex and potential interactions provide insights into the inhibitory role of the ligand.

## 3 Results and discussion

### 3.1 Dose-response evaluation of arginine and ZnCl_2_ on MS2 bacteriophage

When arginine was used independently on MS2 at concentrations ranging from 0.5 to 5%, it failed to demonstrate any virucidal efficacy. However, a synergistic combination of arginine and ZnCl_2_ revealed a notable dose-dependent virucidal effect. Specifically, the mixture of 2% arginine with 0.1% ZnCl_2_ at pH 10 exhibited robust virucidal activity, with a 3.47 log reduction (Table 1). pH 10 was selected based on efficacy testing across acidic, neutral, and alkaline pH levels, where only the alkaline conditions demonstrated efficacy (data not shown). Thus, this formulation was selected for further testing against FCV.

**TABLE 1:** Long reduction of MS2 after 10 min exposure to various concentrations arginine and ZnCl_2_.

Arginine (%)	ZnCl_2_ (%)	Average log R of MS2	SD
0.5%	0.00%	0.17	0.27
1.0%	0.00%	0.20	0.16
2.0%	0.00%	0.25	0.14
3.0%	0.00%	0.14	0.06
4.0%	0.00%	0.31	0.12
5.0%	0.00%	0.19	0.02
0.5%	0.01%	1.76	1.36
1.0%	0.01%	0.97	0.14
2.0%	0.01%	0.95	0.13
3.0%	0.01%	1.48	0.69
4.0%	0.01%	1.35	0.47
5.0%	0.01%	1.16	0.22
0.5%	0.05%	0.93	0.24
1.0%	0.05%	2.60	0.03
2.0%	0.05%	2.83	0.08
3.0%	0.05%	3.00	0.47
4.0%	0.05%	2.58	0.14
5.0%	0.05%	2.72	0.05
0.5%	0.10%	0.47	0.56
1.0%	0.10%	1.32	0.08
2.0%	0.10%	3.47	0.03
3.0%	0.10%	3.14	0.04
4.0%	0.10%	2.84	0.25
5.0%	0.10%	3.35	0.57

The combination of 2% arginine and 0.1% ZnCl_2_ presents the minimal concentration which achieves the highest log reduction of MS2, which was selected for future studies.

### 3.2 Virucidal efficacy of 2% arginine with 0.1% ZnCl_2_ against FCV

Feline calicivirus was subjected to formulations comprising 2% arginine, 0.1% ZnCl_2_, and a combination of both ingredients for a duration of 10 min, according to BS EN 14476:2013+A2:2019 quantitative suspension test methods. Recognizing the potential impact of pH on the efficacy of the arginine and ZnCl_2_, a range of alkaline pH levels from pH 9–11 was included.

**FIGURE 1:**
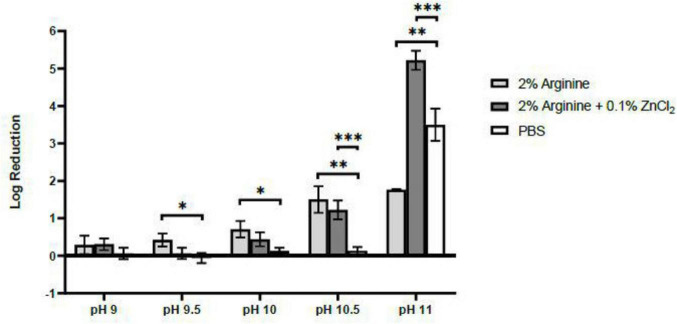
Virucidal Efficacy of 2% arginine and 2% arginine + 0.1% ZnCl_2_ in various alkaline pH against feline calicivirus (FCV) after 10 min. Although both 2% arginine and 2% arginine + 0.1% ZnCl_2_ both showed increasing virucidal efficacy as pH increases from 9 to 11, a synergistic effect between arginine and ZnCl_2_ was only observed from pH 10.5 and pH 11. However, at pH 11, a pH effect against FCV was observed from the negative control, PBS, contributing to a 3.5 log reduction. Statistical analysis of differences in log reduction of FCV was performed with unpaired *t*-test: *p < 0.05, **p < 0.01, ***p < 0.005.

The virucidal effect of the single ingredient, 2% arginine, against FCV strengthened with an increase in pH, peaking at a 1.76 log reduction at pH 11.5. However, this effect was lower than the recommended minimum 4 log reduction from regulatory bodies. Nonetheless, at pH 11, the combined formulation of 2% arginine and 0.1% ZnCl_2_ demonstrated a synergistic effect, achieving a 5.22 log reduction of FCV, surpassing the requirement threshold for virucidal efficacy claims (Figure 1).

A control setup consisting solely of ZnCl_2_ was not prepared due to the chemical reaction that occurs at alkaline pH, where ZnCl_2_ reacts with sodium hydroxide, resulting in the formation of a precipitate of zinc hydroxide. Given that all reagents were pre-filtered through a 0.2 μm filter to ensure sterility, this process removed most of the insoluble zinc compounds, rendering it an unsuitable control for our study. Therefore, PBS was used as a replacement and interestingly at pH 11, PBS exhibited a 3.50 log reduction in virucidal effect against FCV within the 10 min contact period (Figure 1). Therefore, to precisely assess the virucidal efficacy attributable to arginine and ZnCl_2_ combination, FCV was exposed to the various formulations at pH 9–11 for shorter durations. This approach aimed to identify only the direct virucidal effects from arginine and ZnCl_2_.

**FIGURE 2:**
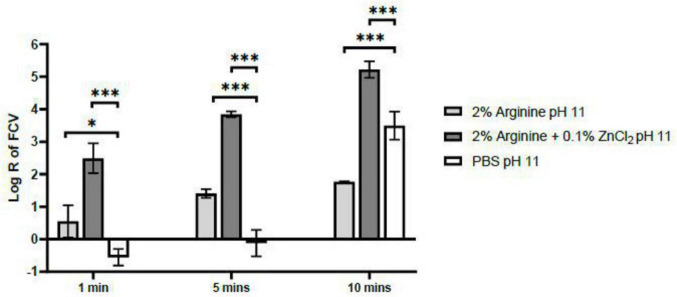
Virucidal Efficacy of 2% arginine and 2% arginine + 0.1% ZnCl_2_ at pH 11 against feline calicivirus (FCV) after 1, 5 and 10 min. While no pH effect on FCV was observed at the 1 min contact time, the log reduction achieved by the combination of 2% arginine + 0.1% ZnCl_2_ was only 2.5, falling short of the minimum requirement for virucidal claims. In contrast, extending the contact time to 5 min resulted in a log reduction of 3.8, without any observed pH influence on the virus. Therefore, a contact time of 5 min is recommended for the 2% arginine + 0.1% ZnCl_2_ combination at pH 11 to ensure effective virucidal activity. Statistical analysis of differences in log reduction of FCV was performed with unpaired *t*-test: *p < 0.05), **p < 0.01, ***p < 0.005.

Shorter contact times of both 1 and 5 min with PBS ceased to exhibit a pH effect on FCV, indicating the exclusion of pH influence within these durations. Moreover, a consistent synergy was observed between arginine and ZnCl_2_, where their combined application consistently outperformed the use of arginine alone in terms of virucidal efficacy (Figure 2). A contact time of 5 min was established in this study as the optimal duration for arginine and ZnCl_2_ combination to exert its virucidal activity against FCV. This determination was based on the absence of a pH effect on FCV and this duration allowed for a higher log reduction of FCV to 3.84, compared to a log reduction of only 2.49 after the 1 min contact time. Although the log reduction of FCV by arginine and ZnCl_2_ combination did not achieve the recommended log reduction of 4 after 5 min, the obtained log reduction is sufficiently close, clearly indicating a synergistic interaction between arginine and ZnCl_2_. Subsequently, the mechanism of action behind this potentiated efficacy was studied.

### 3.3 Impact of pH on the stability, reversibility, and FCV interaction of the Arg-Zn^2+^-Arg complex

**FIGURE 3:**
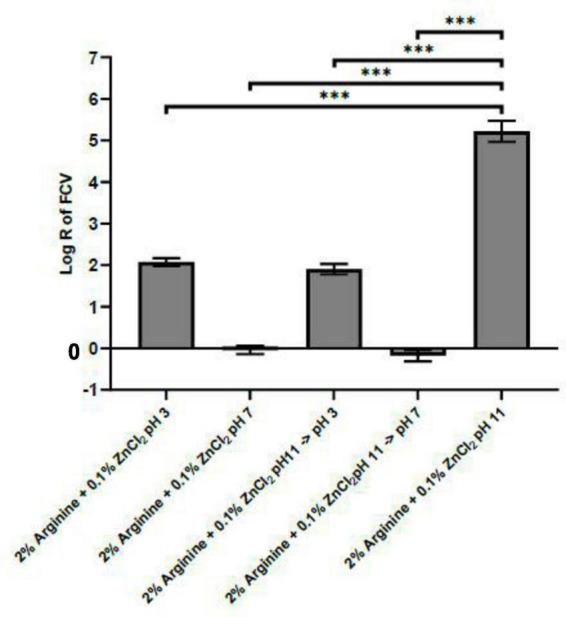
pH impact on stability of Arg-Zn^2+^-Arg complex. The Arg-Zn^2+^-Arg complex was initially allowed to form at pH 11, after which the pH was adjusted to pH 3 and 7 to assess the stability of the complex or its potential for dissociation. The complex dissociates under these altered pH conditions, as evidenced by the diminished virucidal efficacy against feline calicivirus (FCV). This suggests that the integrity and virucidal activity of the Arg-Zn^2+^-Arg complex are significantly influenced by the pH environment, with stability at pH 11 not being maintained upon shifting to more acidic or neutral pH levels. Statistical analysis of differences in log reduction of FCV was performed with unpaired *t*-test: *p < 0.05, **p < 0.01, ***p < 0.005.

Our experimental data indicated that the complex formed by arginine and ZnCl_2_ exhibits optimal efficacy at pH 11, prompting an investigation into the stability of this complex when the pH deviates from this optimal point. We aimed to determine if a shift in pH away from 11 would lead to the dissociation of the complex and whether the virucidal effect on FCV caused by the Arg-Zn^2+^-Arg complex is reversible with changes in pH. To explore these questions, the complex was allowed to form at pH 11, subsequently adjusting the pH to 3 and 7, levels at which the arginine and zinc combination was shown to be ineffective against FCV. Our findings suggest that the Arg-Zn^2+^-Arg complex could potentially dissociate when the formulation’s pH is altered from the optimal pH 11, as evidenced by the loss of virucidal efficacy at these altered pH levels, depicted in Figure 3. There is limited literature data concerning the stoichiometry of arginine and zinc. However, existing literature on the interaction between lysine and zinc typically indicates a 2:1 stoichiometry (Conato et al., 2000; Leu, 1990). Given that both lysine and arginine are basic amino acids, this study assumes a similar 2:1 stoichiometry for arginine and zinc. Consequently, all subsequent modeling and docking studies was conducted based on this stoichiometry.

**FIGURE 4:**
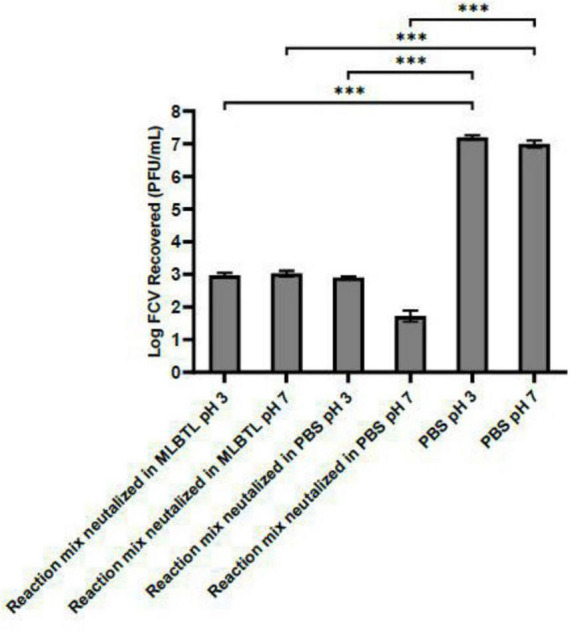
pH impact on interaction between Arg-Zn^2+^-Arg complex and feline calicivirus (FCV) following initial contact. The Arg-Zn^2+^-Arg complex was allowed to form and subsequently interacted with FCV at pH 11 for a duration of 5 min. Following this interaction, the reaction mixture was neutralized, and the pH was adjusted to pH 3 and 7. The concentration of FCV recovered from the reaction mixture was significantly lower compared to the PBS negative control. This indicates that upon interaction, the effect of the complex on FCV is not reversible by alterations in pH. Statistical analysis of differences in log reduction of FCV was performed with unpaired *t*-test: *p < 0.05, **p < 0.01, ***p < 0.005.

Furthermore, Figure 4 demonstrates that when the reaction mixture of the virus and Arg-Zn^2+^-Arg complex was neutralized to pH 3 and 7 using MLBTL after 5 min, the amount of virus recovered was 4 log lower compared to PBS negative controls. This finding suggests that the impact of the Arg-Zn^2+^-Arg complex on FCV remains unaffected by pH changes after the complex has interacted with the virus.

### 3.4 Investigation of Arg-Zn^2+^-Arg mechanism of action (MoA) on FCV using DLS and TEM

**FIGURE 5:**
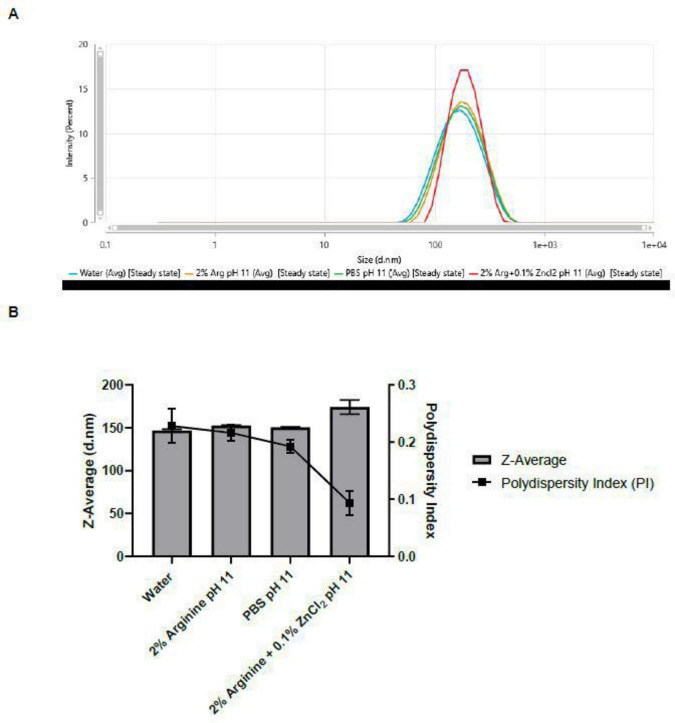
Dynamic light scattering (DLS) analysis of feline calicivirus (FCV) after treatment with Arg-Zn^2+^-Arg complex at pH 11 for 5 min. **(A)** Size diameter distribution by intensity of FCV **(B)** Hydrodynamic Diameter (Z-Average) and polydispersity index (PI) of FCV. A 5 min treatment with the Arg-Zn^2+^-Arg complex at pH 11 revealed minimal changes in the size of the virus particles. This observation suggests that the potential MoA of the Arg-Zn^2+^-Arg complex does not involve the disruption of the viral capsid. Typically, a disruption mechanism would result in a decrease in the average size of the viral particles, reflected by a shift to the left of the size diameter distribution in the intensity graph. DLS analysis indicates that the complex affects the virus through a different mechanism.

Dynamic light scattering data indicated that the interaction between FCV and the Arg-Zn^2+^-Arg complex does not result in a significant change in the virus size (Figure 5). This observation implies the possibility that like most viruses, FCV particles naturally aggregate without external influence as a defensive mechanism and to increase the multiplicity of infection (MOI) to enhance the probability of successful infection (Pradhan et al., 2022). Consequently, these observations suggest that the MoA of Arg-Zn^2+^-Arg complex against FCV likely does not involve the disruption of the viral capsid.

**FIGURE 6:**
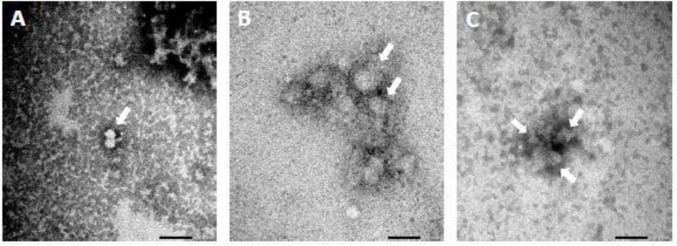
Transmission electron microscopy (TEM) of feline calicivirus (FCV) after 5 min treatment with **(A)** PBS at pH 11 **(B)** 2% arginine at pH 11 **(C)** 2% arginine + 0.1% ZnCl_2_ at pH 11. Scale bar = 100 nm. TEM images show that FCV form clusters after treatment but still retained structural integrity. This further suggest that the Arg-Zn^2+^-Arg complex utilizes a non-capsid disrupting antiviral mechanism of action (MoA).

Transmission electron microscopy analysis of FCV subjected to 2% arginine treatment revealed clustering of the viral particles, while their structural integrity remained unaffected. In contrast, when FCV was challenged with a combination of 2% arginine and 0.1% ZnCl_2_, it presented darker clusters of FCV compared to those treated with 2% arginine alone and the PBS control. This darkening is likely a result of the test compounds reacting with the PTA staining. However, upon examination it was shown that the FCV still maintained its structural integrity (Figure 6). These observations are in line with the DLS data, which indicated that FCV capsid was undisturbed. This again suggests that the potential MoA of Arg-Zn^2+^-Arg complex does not involve disruption of the viral capsid.

### 3.5 Molecular modeling and docking studies

To identify potential areas on the FCV capsid for Arg-Zn^2+^-Arg ligand binding, we carried out binding pocket predictions. The highest-ranked pocket was selected for molecular docking calculations to elucidate the interactions facilitating the binding of the Arg-Zn^2+^-Arg ligand to the FCV capsid.

**FIGURE 7:**
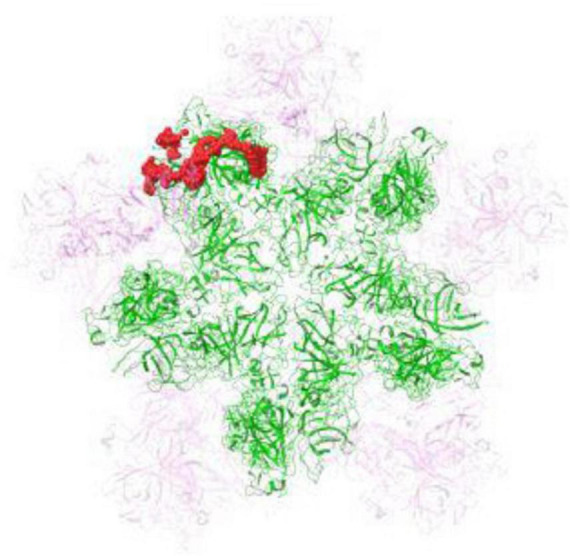
Structural representation of the feline calicivirus (FCV) capsid 5-fold vertex model. FCV pentamer prepared for binding site analysis (presented in green), with adjacent capsid proteins in the viral shell (in semi- transparent magenta). The most druggable binding pocket, critical for ligand interaction, is accentuated as red spheres, denoting the region where the Arg-Zn^2+^-Arg complex is predicted to bind.

The FCV capsid, featuring icosahedral symmetry, primarily consists of VP1 protein dimers. These dimers assemble into 90 units, organized as various protomers, to form the protective capsid encasing the viral RNA (Makino et al., 2006). In this study, binding pocket prediction and molecular docking calculations were performed on the pentameric capsid shell assembly (Figure 7).

Our molecular docking study illuminates the specific interactions between the Arg-Zn^2+^-Arg complex and the FCV capsid, notably within a binding pocket crucial for fJAM receptor engagement. The identification of this pocket as the primary interaction site highlights the Arg-Zn^2+^-Arg ligand’s potential to inhibit FCV by mimicking the natural receptor’s binding mechanism, thus preventing viral entry.

**FIGURE 8:**
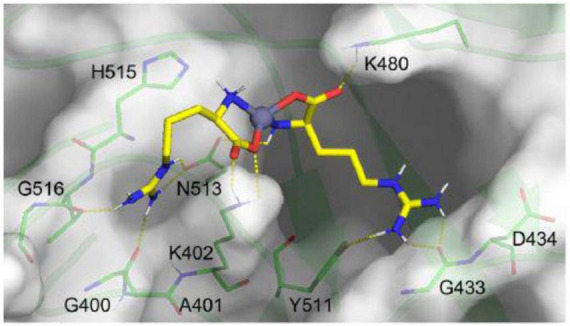
Molecular interaction of Arg-Zn^2+^-Arg ligand within key binding pocket of feline calicivirus (FCV) capsid. Illustrated in yellow sticks, the ligand engages the top-ranked pocket (transparent surface) of the FCV capsid. Yellow dashes highlight hydrogen bonds between the ligand and key residues (green sticks), showcasing its potential to block fJAM receptor binding.

Presented in Figure 8, molecular docking simulations revealed that the Arg-Zn^2+^-Arg ligand forms stable hydrogen bonds with key FCV capsid residues, such as K402, K480, Y511, N513 (side chains), and G516, G400, G433 (backbone), crucial for the virus’s infectivity and receptor interaction (Lu et al., 2018). Such a binding mode of the Arg-Zn^2+^-Arg complex potentially disrupts FCV’s ability to infect host cells and underscores the strategic significance of these interactions in designing antiviral strategies.

The findings corroborate the critical role of the JAM receptor in FCV infectivity, emphasizing the identified binding pocket as a strategic antiviral target (Bhella and Goodfellow, 2011; Ossiboff and Parker, 2007). The Arg-Zn^2+^-Arg ligand likely blocks the binding of the fJAM receptor by occupying the same binding site, interacting with key conserved residues known to facilitate fJAM receptor engagement. This binding mimicry potentially diminishes viral infectivity by preventing the necessary viral-host cell interface formation.

The MoA elucidated through our modeling study differs from previous literature, which primarily emphasized the antibacterial efficacy of the arginine and zinc combination. These prior studies demonstrated how this combination neutralizes bacterial pathogenicity, enhances cell barrier functionality, and disrupts the integrity of bacterial biofilms (Ben Lagha et al., 2020; Gloag et al., 2022). In contrast, our research reveals a novel antiviral property of the arginine and ZnCl_2_ combination, showcasing its capability to inhibit interactions between viruses and host cells, thereby preventing viral entry. Despite these differences in action against bacteria versus viruses, both our study and preceding publications converge on a critical point: the arginine and zinc combination exhibit its antibacterial and antiviral effectiveness exclusively in alkaline pH conditions. This shared characteristic underscores the importance of pH in the application of arginine and zinc, regardless of the microbial target, which could be attributed to the specific stoichiometric relationship between arginine and zinc.

## 4 Conclusion

Our findings reveal a novel synergistic virucidal effect of arginine and ZnCl_2_ at an alkaline pH, specifically pH 11, a departure from previous research which primarily highlighted bactericidal properties. The formation of the Arg-Zn^2+^-Arg complex is pH-dependent, yet its virucidal impact on FCV remains stable across pH variations, underscoring the importance of maintaining optimal pH conditions to ensure consistent efficacy over the product’s shelf life. A limitation encountered in our study was the observed pH effect of our PBS negative control on FCV at a 10 min contact time. We addressed this by reducing the contact time, but future studies may benefit from employing a different buffer system better suited for high alkalinity conditions. This adjustment could potentially allow for the extension of contact times beyond 5 min, thereby facilitating the Arg-Zn^2+^-Arg complex to achieve a log reduction greater than four, as recommended by international guidelines.

Furthermore, our study also successfully demonstrated that MS2 serves as an effective surrogate for FCV, significantly reducing the time required to identify the optimal concentration of the arginine and ZnCl_2_ combination for virucidal activity. The rapid virucidal efficacy results against MS2, available within 24 h, contrast with the longer duration needed for mammalian virus plaque assays, which require at least 3 days for plaque enumeration.

Lastly, this research is among the pioneering efforts to utilize DLS for predicting MoA of virucidal agents. Our data suggests that the Arg-Zn^2+^-Arg complex’s MoA against FCV does not involve capsid disruption, a hypothesis supported by electron microscopy and predictive structural modeling. The latter suggests that the Arg-Zn^2+^-Arg complex might inhibit FCV’s interaction with the host cell’s fJAM receptor. Future work could further investigate this proposed MoA through early viral entry assays on FCV and other viruses of the *Caliciviridae* family, offering deeper insights into the complex’s virucidal dynamics. Nonetheless, the specificity of these interactions highlights the potential of such molecular mimicry in future antiviral strategies, underscoring the efficacy of targeting viral-host interaction sites to efficiently thwart viral infectivity.

## Data Availability

The datasets presented in this study can be found in online repositories. The names of the repository/repositories and accession number(s) can be found in the article/Supplementary material.
