# Supplementary figures and images for: Exploring non-alcohol-based disinfectant: virucidal efficacy of arginine and Zinc chloride against feline calicivirus
Source: Front Microbiol. 2025 Feb 13;16:1550295. doi: 10.3389/fmicb.2025.1550295 (PMC11865247; doi:10.3389/fmicb.2025.1550295)

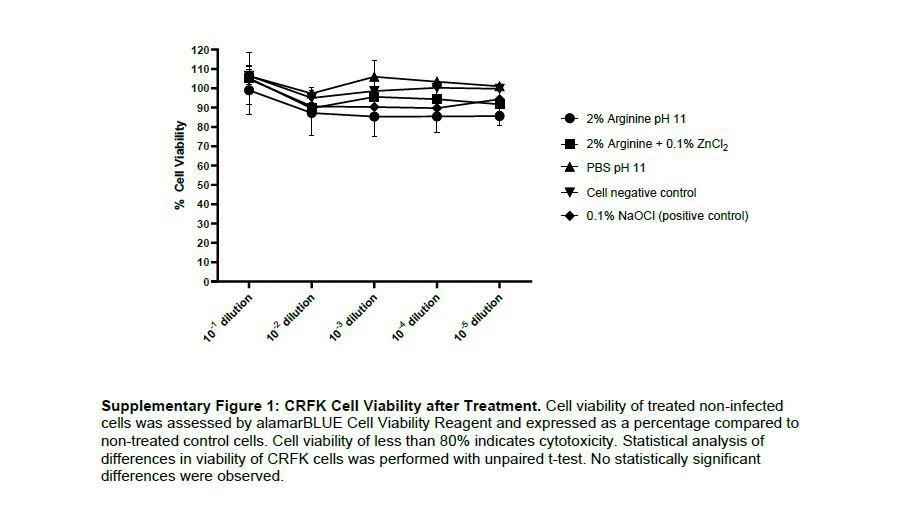

Supplement: Supplementary file 1 [file Image_1.JPEG]
